# The Amalgam Question

**Published:** 1897-12

**Authors:** J. N. Crouse

**Affiliations:** Chicago, Ills.


### The Amalgam Question.

BY J. N. CROUSE, D.D.S., CHICAGO, ILLS.

Abstract of paper read before the American Dental Association, Old Point Comfort, Va., August, 1897.

There are elements of uncertainty about amalgam and its use in dentistry which are more than trivial. Whether performed thoroughly or in the most careless manner, fillings of amalgam are far from satisfactory. What is the stimulus to put forth our best efforts in the use of a second-best filling material, when out of more than sixty different preparations, accurately tested, not one met all the requirements for a good filling material? We are indebted to Dr. G. V. Black for the first scientific method of testing amalgam, but the average dentist is without any means of determining the quality or behavior of the material that is more used than all others combined, It is entirely guess-work in making a selection without an elaborate and expensive outfit and the expenditure of much time in careful work. A common fallacy in the selection of an amalgam is that it must be very plastic and easily mixed. This is a great mistake, as it is impossible to pack it perfectly in a cavity. Another mistaken idea is that it is injured by manipulation after it commences to set. The exact opposite is true, for a stronger and better mass can be made if the amalgam be put to place by heavy hand pressure or by malleting after it has fairly begun to set.

A third fallacy is that the least amount of mercury possible to make the mass pliable is the correct manner of mixing, but in this case again the opposite is true.

Having brought with me a micrometer and dynamometers, and other implements, I invite inspection and practical tests. Many essential qualities can be shown and great benefits gained by taking part in this work.

